# Supplementary material for: DMRforPairs: identifying Differentially Methylated Regions between unique samples using array based methylation profiles
Source: BMC Bioinformatics. 2014 May 15;15:141. doi: 10.1186/1471-2105-15-141 (PMC4046028; doi:10.1186/1471-2105-15-141)
Supplement: Additional file 2 — DMRforPairs output for the comparison of A431-MCF7 and NA17018-NA17105. Please start from the HTML files in each folder. Available via the BMC Bioinformatics website. [file 1471-2105-15-141-S2.zip › 1394847754114233_MOESM2_ESM/A431_MCF7/figures/32356.pdf]

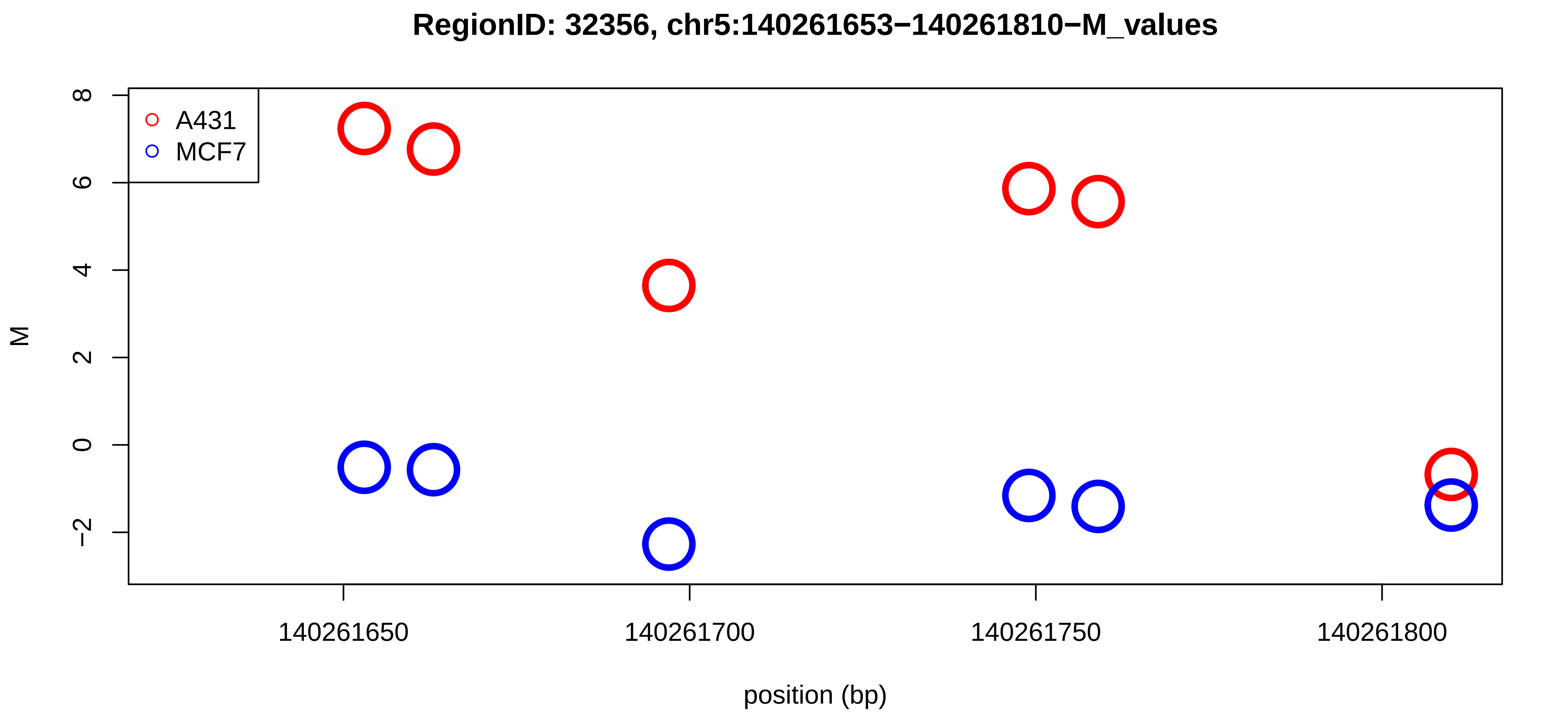
RegionID: 32356, chr5:140261653−140261810−M_values
A431
MCF7
M
8
6
4
2
0
−2
140261650
140261700
140261750
140261800
position (bp)

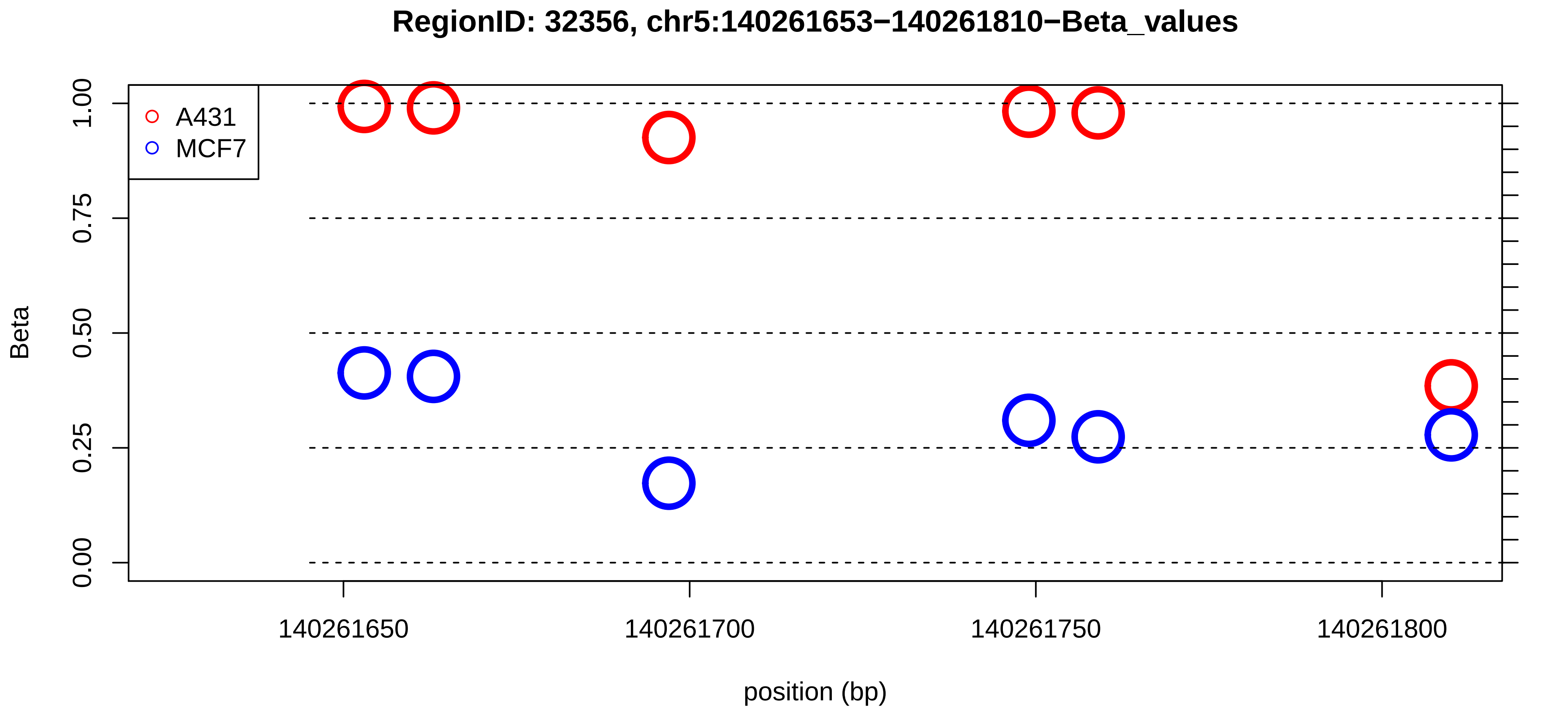
RegionID: 32356, chr5:140261653−140261810−Beta_values
A431
MCF7
Beta
1.00
0.75
0.50
0.25
0.00
140261650
140261700
140261750
140261800
position (bp)

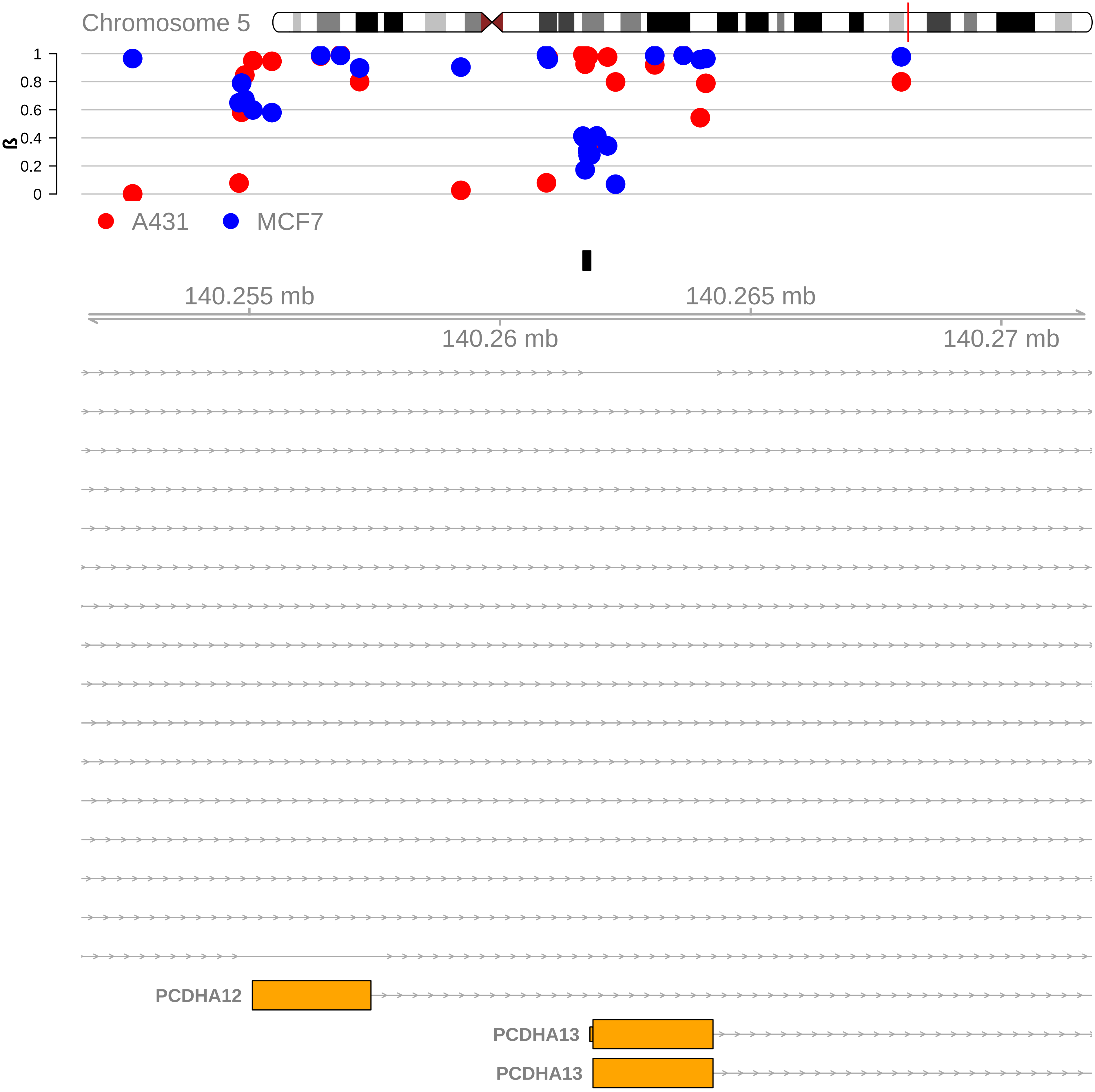

Chromosome 5
ß
1
0.8
0.6
0.4
0.2
0
A431
MCF7
140.255 mb
140.265 mb
140.26 mb
140.27 mb
PCDHA12
PCDHA13
PCDHA13
